# Research Attitude and Interest among Cancer Survivors with or without Cognitive Impairment

**DOI:** 10.3390/cancers15133409

**Published:** 2023-06-29

**Authors:** Ding Quan Ng, Daniella Chan, Munjal M. Acharya, Joshua D. Grill, Alexandre Chan

**Affiliations:** 1 School of Pharmacy and Pharmaceutical Sciences, University of California Irvine, Irvine, CA 92697, USA; 2 School of Medicine, University of California Irvine, Irvine, CA 92697, USA; 3 School of Biological Sciences, University of California Irvine, Irvine, CA 92697, USA

**Keywords:** research attitudes, research interests, willingness to participate, cognition, cancer cancer-related cognitive impairment

## Abstract

**Simple Summary:**

Currently, there remains a lack of interventions which sufficiently address the management of cancer-related cognitive impairment (CRCI). Therefore, this study focused on determining the research attitudes and interests of cancer survivors affected by cognitive impairment to improve the understanding of their interest in various clinical research procedures and design studies sought by these survivors. Among cancer survivors registered under the University of California Irvine Consent-to-Contact registry, those with perceived cognitive impairment were more interested in research involving approved medications, lumbar punctures, and autopsies compared to those experiencing less cognitive symptoms. Such results can serve to benefit the facilitation of the pathogenesis and monitoring of CRCI, as this study brings light to the consideration of research methods that are traditionally less utilized.

**Abstract:**

Background: We examined the research attitudes and willingness to participate in clinical research among cancer survivors with varying degrees of cognitive function. Methods: This is a secondary analysis of data collected through the University of California Irvine Consent-to-Contact registry. Cancer survivors completed the Cognitive Function Instrument (CFI), the Research Attitudes Questionnaire (RAQ), and willingness to participate (WTP) in certain research procedures. Perceived cognitive impairment (CI) was defined as the worst 20% CFI scores. Results: Here, 265 CI and 909 cognitively non-impaired (CNI) participants’ data were analyzed. Mean age and sex distribution were similar, with fewer non-Hispanic Whites and education years among CI participants. More CI participants self-reported past diagnoses of Alzheimer’s disease, mild cognitive impairment, stroke, depression, post-traumatic stress disorder, and alcohol abuse (all *p* < 0.05). CI participants were significantly more interested in studies investigating approved medications (92% vs. 87%, *p* = 0.030), lumbar puncture (47% vs. 38%, *p* = 0.027), and autopsy (78% vs. 69%, *p* = 0.022). After removing survivors with co-existing neuropsychiatric conditions, interest in autopsy studies remained statistically higher among CI (79% vs. 69%, *p* = 0.022). Conclusions: Participants with cancer and CI are open to research procedures and interventions that are traditionally less utilized, which may facilitate the discovery of the pathogenesis and interventions for cancer-related cognitive impairment (CRCI).

## 1. Introduction

Cancer-related cognitive impairment (CRCI) refers to the decline in cognitive abilities experienced by cancer survivors of all ages. Symptoms are presented as difficulties with memory, executive function, processing speed, and attention, leading to challenges in the pursuit of higher education, management of personal finances, and fulfilling family and work responsibilities [1,2,3]. Causes of CRCI have been documented to be multifactorial in nature, and hence it remains a significant quality of life issue and an unmet need among cancer survivors due to its limited management options [4,5,6].

To address such unmet need, research generating quality data from observational and interventional studies in cancer survivors with CRCI will be critical. Understanding patients’ attitudes and willingness to participate in research allows researchers to design studies that are sought by patients, which may improve patients’ willingness to participate and adhere to study procedures. Past studies have shown that improvement of symptoms was a motivation for patients with advanced cancer to participate, while perceived risks of symptom worsening was a barrier [7]. In non-cancer populations, those with worse cognitive function were also more likely to participate in higher-risk studies that seek to prevent or delay further impairments [8,9]. These studies, however, did not focus on cognitive impairment in cancer populations, leaving a knowledge gap regarding the level of research-associated risks that CRCI-afflicted survivors would be willing to bear to participate in clinical research.

For this study, we take a hypothesis-generating approach to compare: (i) the differences in attitudes towards research and (ii) the types of interested research activities (interventions and study procedures) between cancer survivors with varying degrees of perceived cognitive function. This study utilizes an existing patient registry that is based in Southern California for recruiting participants interested in various research studies [10]. By engaging with cancer survivors and understanding their perspectives, we can ensure that research in this area is more patient-centered and that results are more meaningful to the individuals affected by CRCI.

## 2. Methods

### 2.1. Data Source and Study Population

The University of California Irvine (UCI) Consent-to-Contact (C2C) online registry was launched in August 2016 to facilitate recruitment into clinical studies and trials (UCI IRB #2015-2494) [10]. Participants were recruited from Orange County, CA, and were at least 18 years of age. Upon registration and completion of informed consent (https://c2c.uci.edu, accessed on 26 June 2023), participants completed electronic cross-sectional questionnaires inquiring about perceived cognitive performance, sociodemographic and clinical characteristics, research willingness, and research attitudes. Since the study-associated risks for the registry are minimal (i.e., unwanted contact by research coordinators for studies relying on the C2C registry for recruitment), assessment of capacity to consent is not required. While the registry was developed by Alzheimer’s disease researchers [10], it was marketed to potential participants using messaging related to assisting with the recruitment of all types of clinical research. Leadership of the registry has also made it available to enhance recruitment to clinical researchers campuswide. Hence, we believe this dataset is suitable to answer non-Alzheimer’s disease types of research questions (e.g., CRCI). For this study, participants who have self-reported a past diagnosis of cancer were included in the analysis.

### 2.2. Sociodemographic, Clinical, and Other Characteristics

Collected sociodemographic information included self-reported race/ethnicity, age, sex, and years of education. Clinical characteristics included self-reported medical history regarding cancer, neurological, and psychiatric diseases, as well as cancer treatment modalities and date of last cancer treatment. Using this information, we identified participants who were receiving active cancer treatment at the time of the survey. Finally, participants expressed their willingness to be notified about research studies conducted at the university campus (Irvine, CA, USA) and the affiliated medical center (Orange, CA, USA).

### 2.3. Assessment of Perceived Cognitive Function

Assignment to either the cognitively impaired (CI) or cognitively non-impaired (CNI) groups was determined by the 14-item Cognitive Function Instrument (CFI). A summary score, ranging from 0 to 14, was computed and weighted by the number of missing CFI responses for each participant, with higher scores indicating worse perceived cognitive function compared to 1 year ago [10,11]. Concurrent validity was reported with objective cognitive function and decline [11,12]. Following current literature on CRCI prevalence [13], the top 20% CFI scores within the sample were classified under the CI group, while the remaining were placed into the CNI group.

### 2.4. Outcomes

To achieve our study objectives, we compared the following outcomes between participants in the CI and CNI groups:

(1). Research attitudes: Participants’ research attitudes were evaluated using a 7-item Research Attitudes Questionnaire (RAQ) [14]. Each item uses a 5-point Likert scale to assess participant agreement: (1) strongly disagree, (2) disagree, (3) neutral, (4) agree, and (5) strongly agree. A summary score, ranging from 7 to 35, was calculated and weighted against the number of missing RAQ responses for each participant, with higher scores representing more positive research attitudes. Previous studies have demonstrated a relationship between the RAQ score and willingness to participate in research, as well as the likelihood of completion of clinical trials [15,16,17].
(2). Willingness to participate in research activities: Participants were asked about their interest in being notified about studies (yes/no) which investigate approved medication, investigational medicine, diet or lifestyle alteration, blood draws, cognitive testing, magnetic resonance imaging (MRI), positron emission tomography (PET), lumbar puncture, autopsy, and on-site/at-home blood draws for genetic testing and biomarker quantification [10]. A brief layman description for each research activity was also elaborated in the survey to improve their understanding of the question. We assessed the proportions of participants who were interested in each of these research activities.

### 2.5. Missing Data

Adapting the methodology implemented by Pucher et al. [18], imputation of missing data was completed using multiple imputation by chained equations (MICE) [19], creating five imputed datasets for propensity score estimations.

### 2.6. Inverse Probability of Treatment Weighting (IPTW)

An inverse propensity score weighting approach was utilized to minimize the effect of confounding factors (sociodemographic variables, cancer-related characteristics, neuropsychiatric comorbidities, and preferred study locations), all of which are listed in Table 1, to compare the relative interest in research and certain research procedures between the CI and CNI groups [20]. A logistic regression approach, which estimates odds ratio as the effect size, could not be carried out as the binary outcomes (willingness to participate in research activities) were hypothesized to be non-rare (>10%). The log-binomial regression, which estimates risk ratios for non-rare binary outcomes, could not converge when estimating some of the models. The propensity score is the probability of being assigned to the exposed group (i.e., CI), conditional on the confounding factors [21], and was estimated using multiple logistic regression in this study. Restricted cubic smoothing splines with five knots were used to model the relationship between each of the continuous variables (age at survey and years of education) and the log-odds of exposure [20]. Subsequently, each case was assigned a weight based on the inverse of the propensity score for the exposure it was given. To correct for very large or small weights which could destabilize the effect estimates, we stabilized the weights via multiplying them with the marginal probability of the exposure assignment [20,22]. This process was repeated for each of the five imputed datasets, with the resulting average stabilized weights determined as the final weighting variable for generating the weighted cohort to facilitate direct comparisons [18]. A correct propensity score model specification is indicated by a mean of one for stabilized weights [20,22].

### 2.7. Assessment of Covariate Balance

Adhering to the IPTW best practices recommended by Austin and Stuart [20], we computed standardized mean differences (SMD) for each individual confounding factor (including interactions and higher-order moments for continuous variables) and confirmed covariate balance by ensuring that all confounders had a value of less than 0.1 in the weighted cohort. To verify the balance on the entire distribution of continuous covariates, side-by-side boxplots and empirical cumulative distribution functions (CDFs) were used for graphical comparisons between the CI and CNI groups in the weighted cohort.

### 2.8. Statistical Analysis

Prior to IPTW, descriptive statistical analyses were carried out on continuous baseline characteristics using the Mann–Whitney U test due to the non-normality of the data. Regarding categorical characteristics, depending on the proportions of cells with counts of less than 5, Pearson’s chi-squared test (<20%) or Fisher’s exact test (≥20%) were used. Outcomes were compared between the CI and CNI groups in the IPTW-weighted cohort, using the Chi-square test for willingness to participate in different research activities and the Mann–Whitney U test for the RAQ score. Analyses were tested at α = 0.05 and completed using R version 4.2.1 [23].

### 2.9. Sensitivity Analysis

To eliminate the confounding effects underlying neuropsychiatric conditions not indicative of CRCI, we conducted a sensitivity analysis to remove all participants who self-reported at least one of any neuropsychiatric conditions, except major depression which was a known clustering symptom with CRCI [24,25,26]. This was followed by missing data imputation, propensity score estimation, IPTW weighting, covariate balance assessments, and inferential analysis, as described above.

## 3. Results

### 3.1. Demographics and Clinical Characteristics

We identified a total of 1165 participants with cancer, with 256 (22%) in the CI group and 909 (78%) in the CNI group. The participants averaged 66 years old (SD = 11.9) at survey completion, received 17 years of education (SD = 2.7), with majority participants being females (61%) and non-Hispanic White (81%). Majority were also skin cancer survivors (46%) and had a history of cancer-related surgery (73%), with some still receiving active cancer treatment (24%) (Table 1). The CI group, compared to CNI, comprised of less non-Hispanic White participants (75% vs. 83%), received less years of education (mean(SD): 16(3.1) vs. 17(2.5)), and had self-reported proportionally more past diagnoses of neuropsychiatric conditions, namely normal-pressure hydrocephalus, Alzheimer’s disease, mild cognitive impairment, stroke, traumatic brain injury, major depression, post-traumatic stress disorder (PTSD), alcohol abuse, bipolar disorder, and schizophrenia (all *p* < 0.05, Table 1).

### 3.2. IPTW Inferential Analysis Findings

Refer to Supplementary Material S1 for the IPTW diagnostic results for the main cohort analysis.

(1). *Research attitudes*: Following IPTW, the mean RAQ scores between the CI and CNI groups (mean (SD): 28.7(4.13) vs. 28.9(4.27), *p* = 0.460) were comparable (Table 2).
(2). *Willingness to participate in research activities:* After IPTW, more CI participants were willing to participate in studies involving approved medication (92.3% vs. 87.2%, *p* = 0.030), lumbar puncture (46.6% vs. 37.5%, *p* = 0.027), and autopsy (77.9% vs. 68.9%, *p* = 0.022). Statistically similar proportions were found for studies involving investigational drugs and alterations to diet/lifestyle, as well as those with cognitive tests, MRI and PET procedures, blood draws, and cheek swabs (Table 2).

### 3.3. Sensitivity Analysis

Refer to Supplementary Material S2 for detailed findings related to sensitivity analysis. In summary, a total of 927 participants did not report self-reported neuropsychiatric conditions (except major depression), with 157 (16%) remaining in the CI group, and 770 (83%) in the CNI group. As with the main analysis results, attitudes towards research were comparable based on the RAQ score (*p* = 0.151). Interest in autopsy studies remained statistically higher in the CI group (78.6% vs. 69.3%, *p* = 0.022), while interest in studies involving approved medications (92.3% vs. 88.0%, *p* = 0.076) or lumbar puncture (45.0% vs. 37.6%, *p* = 0.096) were only proportionally higher. However, only in the sensitivity analysis was there statistically more interest in providing a blood sample for genetic testing in the CI group compared to CNI participants (98.4% vs. 95.3%, *p* = 0.043).

## 4. Discussion

This study evaluated the effects of cognitive impairment among cancer survivors on willingness to participate in research and research attitudes to identify survivors’ interest in CRCI research. While the attitudes towards research were similar between the groups as per the RAQ scores, those with cognitive impairment demonstrated more interest in research involving approved medications, as well as lumbar puncture and autopsy procedures, falling in line with existing literature suggesting cognitively impaired individuals have a higher willingness to participate in clinical research [8]. Such attitudes would encourage pre-clinical and clinical studies for the development of mechanism-based mitigation strategies addressing this long-term quality of life issue.

Our findings have highlighted that survivors are interested to explore certain innovative modalities for managing their cognitive symptoms (more than four in five cancer survivors with CI). For approved medications, that would involve the process of repurposing currently approved medications, that have shown neurocognitive benefits, and may potentially be useful for managing CRCI. Drug repurposing, also known as drug repositioning or reprofiling, is defined as a process that finds new therapeutic uses for existing drugs different from the original medical indication [27]. Repurposing existing drugs is an effective tool for drug development. As the 1988 Nobel Prize in Medicine James Black simply put, ‘the best way to discover a new drug is to start with an old one’ [28]. More importantly, drug repurposing offers shorter routes to the clinic to address diseases with unmet needs that require effective treatment. A number of potential agents that are approved for other indications, such as riluzole [29] for amyotrophic lateral sclerosis (based on its potential action to augment brain-derived neurotrophic factor (BDNF)) and memantine [30] for dementia (based on modulation of neuroinflammation), are potential approved medications that could be useful to repurpose for CRCI after thorough investigation. However, repurposing of existing drugs may not necessarily be the final solution to finding the cure for CRCI. A number of other drugs, such as epoetin alfa and methylphenidate, have been evaluated for management of CRCI, yet repurposing of these drugs for CRCI was not deemed successful [31], likely because these medications were not targeting specific mechanisms associated with CRCI. Clearly, understanding the underlying mechanisms of CRCI and repurposing drugs that would target specific underlying pathways is an important strategy that scientists need to consider.

Our results have also highlighted that there is more willingness to undergo a lumbar puncture procedure among those with CRCI compared to those without CRCI. Lumbar punctures, or spinal taps, are predominantly utilized to obtain cerebrospinal fluid (CSF) samples, which consist of biomarkers useful for understanding the pathophysiology of neurodegenerative diseases. Particularly, higher levels of certain CSF biomarkers (e.g., total tau and amyloid-β) associated with neuronal damage have been shown to act as an accurate predictor of progression from mild cognitive impairment to Alzheimer’s disease [32]. In cancer, a study indicated greater amounts of the CSF biomarker F2-isoprostane in children with acute lymphocytic leukemia (ALL), correlated with poorer attention and inhibitory control [33]. It does have to be kept in mind, however, that access to the CSF samples in these patients was made feasible by intrathecal chemotherapy, a relatively common treatment administered for ALL, indicating the limited utility of lumbar puncture for routine CRCI diagnosis in those not receiving intrathecal treatment. Moreover, our results revealed a relatively low willingness to participate, with both groups having less than half of the participants responding favorably. Nevertheless, research using CSF samples can still be valuable for validating the accuracy of non-CSF biomarkers, such as peripheral biomarkers obtained from plasma and serum, for monitoring CRCI progression. Specifically, amyloid-β peptide and neurofilament light, common biomarkers used to detect underlying Alzheimer’s pathology, concentrations between CSF and plasma samples both independently predicted a decline in a global cognitive composite [34]. Therefore, in terms of research, questions related to the relationships between peripheral and central nervous system biomarkers could potentially be explored with the incorporation of lumbar puncture procedures to facilitate the validation and qualification of peripheral CRCI biomarkers.

Considering the scarcity of literature regarding interest in autopsy studies among cancer survivors with cognitive issues, our results bring light to the prospective creation of brain banks for facilitating CRCI neuropathological research as a feasible method to enhance understanding of CRCI. Past neuropathological studies in neurodegenerative diseases have helped the respective fields to progress by validating clinical phenotypes with post-mortem brain tissue analyses, enhancing evaluations of genetic mutations’ pathogenesis associated with the diseases (in comparison to animal models), as well as determining the measurement accuracy (sensitivity and specificity) of candidate biomarkers [35,36,37]. Few have utilized neuropathological approaches to investigate CRCI mechanisms, save for two retrospective studies [38,39]. Gibson et al. found that oligodendrocyte expression, a marker of myelination, is markedly decreased in post-mortem tissue samples from the subcortical white matter at the frontal lobe (*n* = 5) compared to non-cancer controls (*n* = 5) [38]. Torre et al. found higher expression of oxidative stress and DNA damage markers, similarly in the frontal lobe, in cancer patients treated with chemotherapy (*n* = 15) compared to non-cancer controls (*n* = 10) [39]. Inevitably, the proliferation of well-designed, prospective neuropathological studies, coupled with longitudinal collection of clinical outcomes and peripheral biomarkers, would benefit the clinical management and research into CRCI, wherein progress has been hindered by a lack of agreement in CRCI phenotypes [40], the dearth of qualified biomarkers for clinical utilization and translational research [41], as well as the confusing link between the lower risk of dementia with a cancer diagnosis [42].

Interestingly, our sensitivity analysis revealed slight differences in the inferential analysis findings when compared to the main analysis. While survivors with CRCI were no longer statistically more willing to participate in studies about approved medications and lumbar puncture, the proportions were still descriptively higher among CI compared to CNI. Further, these proportions remained similar between the main and sensitivity analyses, which is arguably more meaningful when thinking about the feasibility of recruiting patients for such studies. More importantly, it is worth discussing the rationale of conducting this sensitivity analysis, which was to unequivocally identify survivors affected with only CRCI without other co-existing neuropsychiatric conditions. Since it is not the standard-of-care to differentially diagnose CRCI patients from other similar conditions (e.g., dementia) in cancer survivors, such approach might be the strictest possible means for a CRCI-focused analysis. There is some evidence to associate neurocognitive disorders such as Alzheimer’s disease and mild cognitive impairment with cancer and anticancer treatment in survivors. Carriers of *APOE4* were previously found with a greater risk of CRCI and Alzheimer’s disease, which suggests a possible pathophysiological connection between both diseases [43]. We postulate that future studies may also face similar problems and suggest that the research and clinical communities develop a set of best practices for researching and managing CRCI in populations confounded by co-occurring neuropsychiatric diseases.

There are several key considerations when translating our findings. Although our study shed light on potential interventions and research procedures that can be further explored, the research community should further investigate whether the expected results of implementing these procedures could add value to the care of patients with CRCI. One could also argue that cancer survivors with cognitive impairment had greater interest in riskier research procedures that might be related to their impaired decision-making capacity associated with pre-existing neurocognitive disorders. While such argument could not be evaluated in the current study, these patients could also be motivated by feelings of altruism (i.e., helping to assess new treatments for future patients), as well as gaining access to treatments that are currently not available in clinical practice, as inferred from studies in other populations [44,45]. Regardless, proper engagement with the local cancer survivorship community remains important to build research trustworthiness and openly address potential research-associated risks. For cancer survivors with worse neurocognitive problems, taking notes from prior dementia studies, researchers should engage patients and caregivers during study development, evaluate the decision-making capacity to complete the informed consent process, and ensure protection of patients’ interests and values, especially when surrogate consent is necessary [46,47,48].

As this study is a secondary data analysis, the data elements collected may not be ideal for our study question. First, CFI, the primary tool to evaluate cognitive function in the study registry, has not been used in CRCI research, which limits the sensitivity and validity of the CRCI classification utilized in our analysis. Robust surveys that would be more appropriate include the PROMIS Cognitive Function Short-Form 8a or the Functional Assessment of Cancer Therapy–Cognitive Function [49,50]. According to the standards for CRCI research developed by the International Cognition and Cancer Task Force, the gold-standard assessments of cognitive function in cancer require neuropsychological testing (to measure objective cognitive function) in cognitive domains of memory, processing speed, and executive function [51], which are impossible to collect with the self-reported surveys implemented for the registry. However, due to the reported discordance between subjective and objective measures of cognitive function [52], there is a possibility that our results may differ compared to neuropsychological testing-based definitions of CRCI. We were also unable to determine the relative diagnosis ages for cancer and neurocognitive disorders (Alzheimer’s disease or mild cognitive impairment) to specifically exclude patients who were diagnosed with neurocognitive issues prior to the cancer diagnosis. Second, the registry was not specifically developed for cancer-related studies; thus, cancer-specific information such as cancer diagnosis and treatment regimens were not sufficiently collected for more comprehensive analyses. Future registries can consider seeking patients’ consent to access their detailed cancer-related information from cancer registries (e.g., California Cancer Registry). Our findings may also lack external validity considering that the participants who volunteered for the registry are likely to be more interested in research compared to non-participants and considering the higher proportion of non-Hispanic White and female participants relative to the local Southern California population. Finally, the questions related to research activities, specifically on lumbar puncture and PET, which are conceptually less understood by laypersons, may have impacted our research findings. Despite these limitations, this study is significant for its novel evaluation and discussion regarding the varying levels of interest in understudied research procedures and their potential benefits to CRCI research at large, backed by findings from a large sample size of >1000 cancer survivors.

## 5. Conclusions

There remain unmet needs in managing CRCI. In this study, we promisingly found that cancer survivors with perceived cognitive impairment are open to research procedures and interventions that are traditionally less utilized by clinical researchers (e.g., repurposing approved medications, lumbar puncture, and autopsy). Research utilizing these approaches may facilitate progress in understanding the pathogenesis and development of interventions for CRCI.

## Figures and Tables

**Table 1:** Baseline characteristics of the CI and CNI groups among cancer survivors in the original sample.

Variables	CNI (*n* = 909)	CI (*n* = 256)	Total (*n* = 1165)	*p*-Value	SMD ^a^
**CFI**					
Mean	1.61	7.14	2.83	-	-
Min, Max	0.00, 4.00	4.08, 14.00	0.00, 14.00		
**Age at survey**					
Mean (SD)	66.60 (11.36)	64.99 (13.40)	66.25 (11.85)	0.144	0.130
Median (Q1, Q3)	68.00 (61.00, 75.00)	67.00 (55.00, 75.00)	68.00 (60.00, 75.00)		
Missing, n (%)	17 (1.87)	5 (1.95)	22 (1.89)		
**Sex, n (%)**					
Male	364 (40.04)	93 (36.33)	457 (39.23)	0.310	0.077
Female	545 (59.96)	163 (63.67)	708 (60.77)		
Other	0 (0.00)	0 (0.00)	0 (0.00)		
**Race/Ethnicity, n (%)**					
Non-Hispanic White	758 (83.39)	191 (74.61)	949 (81.46)	0.001 **	0.258 ^b^
Hispanic	36 (3.96)	18 (7.03)	54 (4.64)		
Black or African American	10 (1.10)	0 (0.00)	10 (0.86)		
Asian	25 (2.75)	15 (5.86)	40 (3.43)		
More than one population	8 (0.88)	4 (1.56)	12 (1.03)		
Refused	21 (2.31)	12 (4.69)	33 (2.83)		
Others	7 (0.77)	5 (1.95)	12 (1.03)		
Missing	44 (4.84)	11 (4.30)	55 (4.72)		
**Years of education**					
Mean (SD)	16.74 (2.52)	15.99 (3.05)	16.57 (2.66)	<0.001 ***	0.268
Median (Q1, Q3)	16.00 (16.00, 18.00)	16.00 (14.00, 18.00)	16.00 (15.00, 18.00)		
Missing, n (%)	10 (1.10)	3 (1.17)	13 (1.12)		
**Neurological Conditions, n (%)**					
Normal Pressure Hydrocephalus	57 (6.27)	26 (10.16)	83 (7.12)	0.038 *	0.143
Alzheimer’s Disease	13 (1.43)	26 (10.16)	39 (3.35)	<0.001 ***	0.381
Mild Cognitive Impairment	4 (0.44)	27 (10.55)	31 (2.66)	<0.001 ***	0.456
Multiple Sclerosis	17 (1.87)	9 (3.52)	26 (2.23)	0.179	0.103
Seizure Disorder	10 (1.10)	4 (1.56)	14 (1.20)	0.521	0.041
Stroke	7 (0.77)	7 (2.73)	14 (1.20)	0.026 *	0.151
Parkinson’s Disease	8 (0.88)	4 (1.56)	12 (1.03)	0.308	0.063
Traumatic Brain Injury	5 (0.55)	7 (2.73)	12 (1.03)	0.007 **	0.173
**Psychological Conditions, n (%)**					
Major Depression	84 (9.24)	57 (22.27)	141 (12.10)	<0.001 ***	0.368
Post-Traumatic Stress Disorder	33 (3.63)	24 (9.38)	57 (4.89)	<0.001 ***	0.237
Alcohol Abuse	14 (1.54)	11 (4.30)	25 (2.15)	0.012 *	0.166
Bipolar Disorder	5 (0.55)	12 (4.69)	17 (1.46)	<0.001 ***	0.263
Drug Abuse	4 (0.44)	4 (1.56)	8 (0.69)	0.074	0.114
Schizophrenia	1 (0.11)	3 (1.17)	4 (0.34)	0.049 *	0.134
**Types of Cancer, n (%)**					
Skin	432 (47.52)	103 (40.23)	535 (45.92)	0.031 *	0.159
Breast	163 (17.93)	44 (17.19)	207 (17.77)	0.813	0.024
Genitourinary	154 (16.94)	33 (12.89)	187 (16.05)	0.122	0.120
Gynecological	78 (8.58)	29 (11.33)	107 (9.18)	0.236	0.091
Gastrointestinal	54 (5.94)	24 (9.38)	78 (6.70)	0.066	0.129
Blood and bone marrow	49 (5.39)	14 (5.47)	63 (5.41)	1.000	0.002
Head and neck (including thyroid and ocular)	41 (4.51)	13 (5.08)	54 (4.64)	0.737	0.025
Lung	22 (2.42)	11 (4.30)	33 (2.83)	0.173	0.150
Brain and CNS	12 (1.32)	5 (1.95)	17 (1.46)	0.554	0.050
Sarcoma	9 (0.99)	5 (1.95)	14 (1.20)	0.363	0.080
**Cancer Treatment, n (%)**					
Did not receive treatment	55 (6.08)	22 (8.59)	77 (6.61)	0.154	0.099
Currently on treatment	205 (22.75)	69 (26.95)	274 (23.52)	0.149	0.107
Radiation	212 (25.00)	61 (23.83)	273 (23.43)	0.670	0.032
Chemotherapy	190 (22.41)	65 (25.39)	255 (21.89)	0.083	0.132
Surgery	677 (79.83)	176 (68.75)	853 (73.22)	0.236	0.088
**Preferred Study Locations, n (%)**					
Are you willing to hear about studies being conducted at the UCI campus in Irvine?					
Yes	854 (94.47)	242 (94.53)	1096 (94.08)	0.877	0.019
No	50 (5.53)	13 (5.08)	63 (5.41)		
Missing	5 (0.55)	1 (0.39)	6 (0.52)		
Are you willing to hear about studies being conducted at the UCI Medical Center in Orange?					
Yes	802 (89.01)	234 (91.41)	1036 (88.93)	0.135	0.120
No	99 (10.99)	19 (7.42)	118 (10.13)		
Missing	8 (0.88)	3 (1.17)	11 (0.94)		

Abbreviations: CFI—Cognitive Function Instrument; CNS—central nervous system; CI—cognitively impaired; CNI—cognitively non-impaired; Q1—quartile 1; Q3—quartile 3; SD—standard deviation; SMD—standardized mean difference. ^a^ SMD < 0.1 indicates a negligible difference in the mean or prevalence of a covariate between two groups. ^b^ SMD for race and ethnicity was obtained comparing the distribution of non-Hispanic White participants and other races between the groups. * *p* < 0.050, ** *p* < 0.010, *** *p* < 0.001.

**Table 2:** IPTW inferential analysis—research attitudes and willingness to participate in research activities.

Outcomes	Non-IPTW		IPTW with Propensity Scores		*p*-Value ^a^
	CNI (*n* = 909)	CI (*n* = 256)	CNI (*n* = 254.4)	CI (*n* = 896.5)	
**RAQ Score**					
Mean (SD)	28.96 (4.30)	28.64 (4.20)	28.94 (4.27)	28.69 (4.13)	0.460
Median (Q1, Q3)	29.00 (27.00, 32.00)	28.00 (27.00, 32.00)	29.00 (27.00, 32.00)	28.00 (27.00, 32.00)	
**Willingness to participate, n (%)**					
Are you willing to hear about studies that involve taking an approved medication?					
Yes	791 (87.02)	232 (90.63)	221.9 (87.22)	827.3 (92.28)	0.030 *
No	113 (12.43)	23 (8.98)	31.4 (12.34)	62.3 (6.95)	
Missing	5 (0.55)	1 (0.39)	1.1 (0.43)	6.9 (0.77)	
Are you willing to hear about studies that involve taking an investigational medication?					
Yes	720 (79.21)	215 (83.98)	203.1 (79.83)	757.8 (84.53)	0.120
No	178 (19.58)	38 (14.84)	48.6 (19.10)	126.4 (14.10)	
Missing	11 (1.21)	3 (1.17)	2.7 (1.06)	12.3 (1.37)	
Are you willing to hear about studies that involve altering your diet or lifestyle?					
Yes	835 (91.86)	237 (92.58)	233.7 (91.86)	846.5 (94.42)	0.315
No	67 (7.37)	18 (7.03)	18.7 (7.35)	49.3 (5.50)	
Missing	7 (0.77)	1 (0.39)	2.0 (0.79)	0.7 (0.08)	
Are you willing to hear about studies that involve blood draws?					
Yes	845 (92.96)	238 (92.97)	235.3 (92.49)	839.4 (93.63)	0.337
No	57 (6.27)	16 (6.25)	17.4 (6.84)	44.3 (4.94)	
Missing	7 (0.77)	2 (0.78)	1.7 (0.67)	12.8 (1.43)	
Are you willing to hear about studies that involve cognitive testing (tests of memory and thinking)?					
Yes	872 (95.93)	245 (95.70)	244.0 (95.91)	847.6 (94.55)	0.844
No	31 (3.41)	7 (2.73)	8.8 (3.46)	27.1 (3.02)	
Missing	6 (0.66)	4 (1.56)	1.6 (0.63)	21.8 (2.43)	
Are you willing to hear about studies that involve magnetic resonance imaging (MRI, a brain scan that does not involve radiation)?					
Yes	833 (91.64)	244 (95.31)	233.5 (91.78)	847.8 (94.57)	0.157
No	72 (7.92)	11 (4.30)	20.0 (7.86)	41.8 (4.66)	
Missing	4 (0.44)	1 (0.39)	0.9 (0.35)	6.9 (0.77)	
Are you willing to hear about studies that involve positron emission tomography (PET, a brain scan that involves a small amount of radiation)?					
Yes	733 (80.64)	225 (87.89)	206.6 (81.21)	766.4 (85.49)	0.171
No	170 (18.70)	30 (11.72)	46.3 (18.20)	122.7 (13.69)	
Missing	6 (0.66)	1 (0.39)	1.5 (0.59)	7.4 (0.83)	
Are you willing to hear about studies that involve lumbar puncture (also known as a spinal tap)?					
Yes	340 (37.40)	126 (49.22)	95.4 (37.50)	417.4 (46.56)	0.027 *
No	559 (61.50)	129 (50.39)	156.3 (61.44)	472.6 (52.72)	
Missing	10 (1.10)	1 (0.39)	2.7 (1.06)	6.5 (0.73)	
Are you willing to hear about studies that involve autopsy after you die?					
Yes	632 (69.53)	199 (77.73)	175.2 (68.87)	698.7 (77.94)	0.022 *
No	270 (29.71)	56 (21.88)	75.8 (29.80)	191.4 (21.35)	
Missing	7 (0.77)	1 (0.39)	3.4 (1.34)	6.4 (0.71)	
Would you be willing to visit UCI Medical Center in Orange OR the medical school in Irvine to provide a blood sample that can be used to test levels of cells, proteins, or lipids for the sake of better identifying participants for studies?					
Yes	827 (90.98)	236 (92.19)	230.9 (90.76)	826.1 (92.14)	0.801
No	79 (8.69)	20 (7.81)	21.2 (8.33)	70.4 (7.85)	
Missing	3 (0.33)	0 (0.00)	2.3 (0.90)	0.0 (0.00)	
Would you be willing to visit UCI Medical Center in Orange OR the medical school in Irvine to provide a blood sample that can be used to test for genes (DNA) for the sake of better identifying participants for studies?					
Yes	814 (89.55)	232 (90.63)	227.2 (89.31)	817.7 (91.21)	0.523
No	89 (9.79)	22 (8.59)	24.1 (9.47)	72.1 (8.04)	
Missing	6 (0.66)	2 (0.78)	3.1 (1.22)	6.7 (0.75)	
Would you be willing to receive a kit that you could use at home to provide a blood sample or swab of cells from inside your cheek to test for genes (DNA) for the sake of better identifying participants for studies?					
Yes	867 (95.38)	248 (96.88)	242.3 (95.24)	874.0 (97.49)	0.224
No	38 (4.18)	8 (3.13)	10.9 (4.28)	22.5 (2.51)	
Missing	4 (0.44)	0 (0.00)	1.2 (0.47)	0.0 (0.00)	

Abbreviations: CFI—Cognitive Function Instrument; CI—cognitively impaired; CNI—cognitively non-impaired; IPTW—inverse probability of treatment weighting; SD—standard deviation. ^a^ The *p*-values were computed with the IPTW-weighted sample. * *p* < 0.05.

## Data Availability

The data presented in this study are available upon request from the corresponding author. The data are not publicly available due to the protection of the privacy of participants’ personal information.

## Supplementary Materials

The following supporting information can be downloaded at: https://www.mdpi.com/article/10.3390/cancers15133409/s1, Supplementary Material S1: IPTW diagnostic findings for main analysis; Figure S1: Standardized mean differences in the unadjusted (original) and weight (IPTW-weighted) cohorts. Figure S2: Distribution of age and education years between CI and CNI groups. Table S1: Baseline characteristics of the CI and CNI groups after excluding participants who self-reported at least one of any neuropsychiatric conditions (except for major depression), prior to IPTW weighting. Supplementary Material S2: Descriptive statistics and IPTW diagnostic findings for sensitivity analysis; Figure S3: Standardized mean differences in the unadjusted (original) and weighted (IPTW-weighted) cohorts, after excluding participants who self-reported at least one of any neuropsychiatric conditions (except major depression). Figure S4: Distribution of age and education years between CI and CNI groups, after excluding participants who self-reported at least one of any neuropsychiatric conditions (except major depression). Table S2: IPTW inferential analysis—research attitudes and willingness to participate in research activities, after excluding participants who self-reported at least one of any neuropsychiatric conditions (except major depression).
