# Supplementary material for: Research Attitude and Interest among Cancer Survivors with or without Cognitive Impairment
Source: Cancers (Basel). 2023 Jun 29;15(13):3409. doi: 10.3390/cancers15133409 (PMC10340755; doi:10.3390/cancers15133409)
Supplement: Supplementary file 1 [file cancers-15-03409-s001.zip › cancers-2413068-supplementary.pdf]

Supplement of "Research attitude and interest among cancer survivors with or without cognitive impairment" (Ng et al, 2023)

Supplementary Material S1: IPTW diagnostic findings for main analysis

IPTW was successfully at achieving covariate balance on all confounders based on the following metrics:

(1) Diagnostics based on the stabilized weights

The mean stabilized weight was close to one, at 0.988, and ranged from 0.232 to 13.014.

(2) Comparison of means and prevalences in the weighted cohort

Before IPTW, 22 of the 35 confounding variables had an SMD greater than 0.1 (**Table 1**). After IPTW weighting, differences of these 22 variables were eliminated. The highest standardized mean difference (SMD) found was 0.073 for race/ethnicity (non-Hispanic White vs others) in the weighted cohort (**Figure S1**).

(3) Comparison of higher-order moments and interactions

The interactions between the continuous variables (age and years of education), as well as SMDs of the square and cubic terms were compared in both the original unweighted and weighted samples. The square term for age, square and cubic terms of education years, and the interaction between age and years of education in the unweighted sample reported a SMD greater than 0.1. In the weighted sample, these were corrected to be less than 0.1 (**Figure S1**).

(4) Graphical comparisons of the distribution of continuous covariates

Referring to **Figure S2**, for age at survey, the boxplots demonstrate a lower median for respondents in the cognitively impaired (CI) group compared to those in the cognitively non-impaired (CNI) group. After IPTW, the median for both groups appeared nearly equal. Similarly for years of education, the median years for the CNI group was greater than that of the CI. Again, following the IPTW, the median between both groups were nearly equal.

Figure S1: Standardized mean differences in the unadjusted (original) and weighted (IPTW-weighted) cohorts

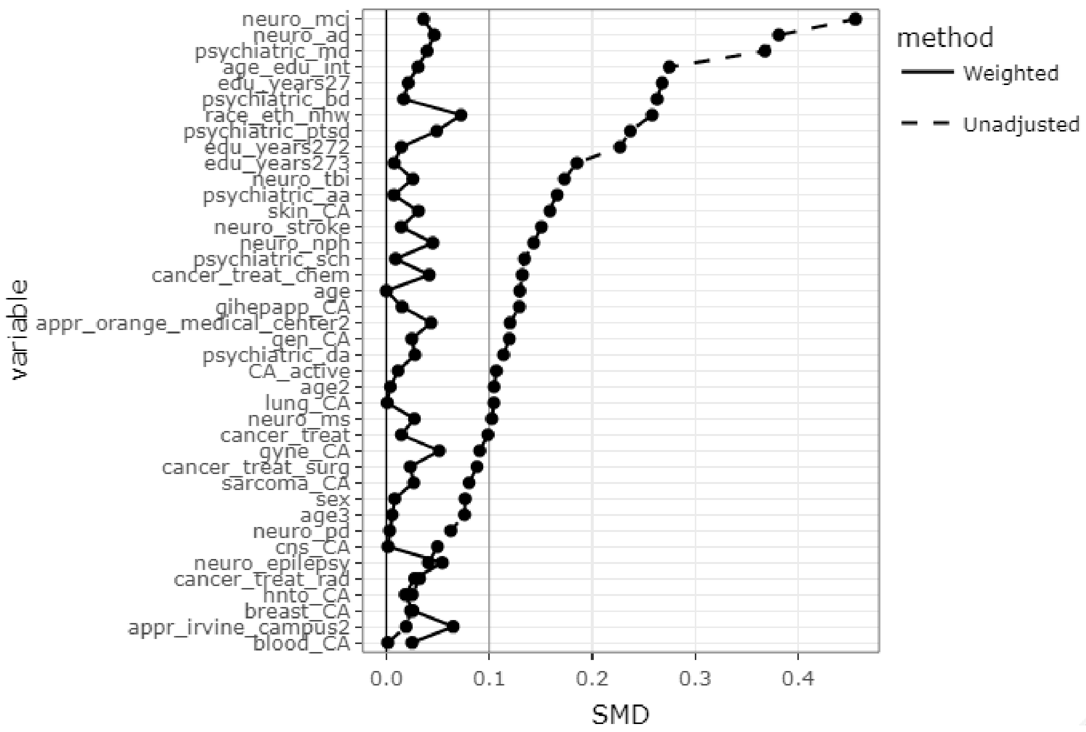

Figure S2: Distribution of age and education years between CI and CNI groups

<table>
<tr><td>Continuous variable</td><td>Unweighted cohort</td><td>Weighted cohort</td></tr>
<tr><td>Age at survey</td><td>
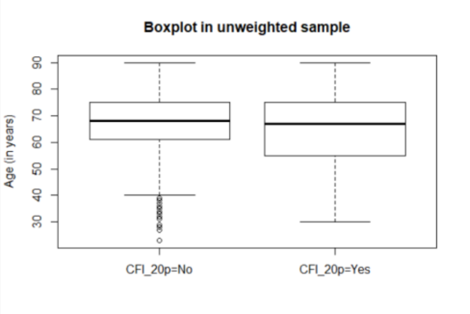

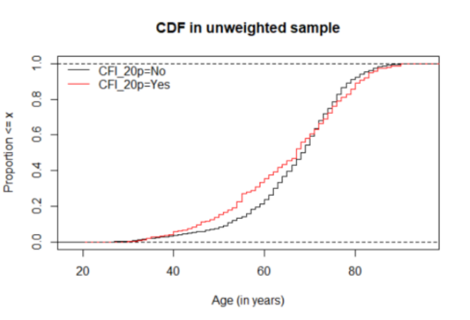

</td><td>
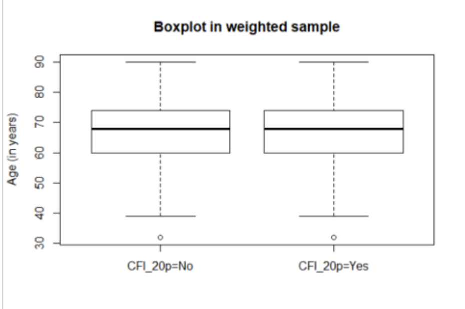

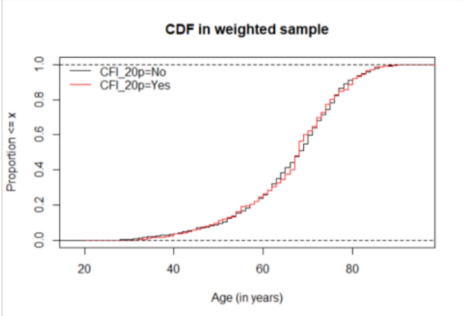

</td></tr>
<tr><td>Years of education</td><td>
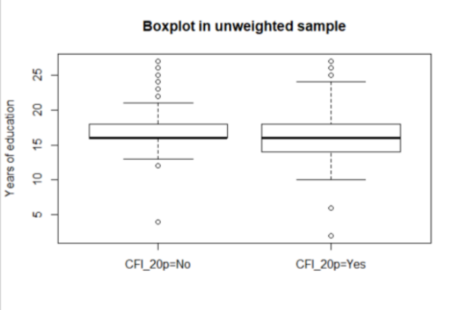

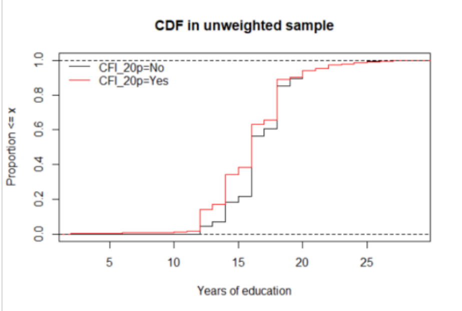

</td><td>
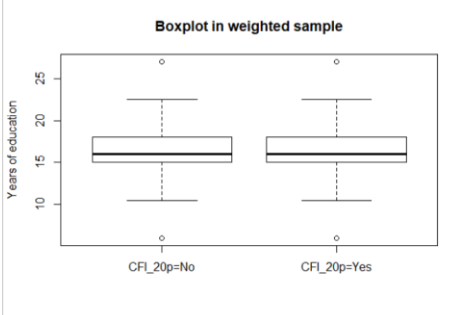

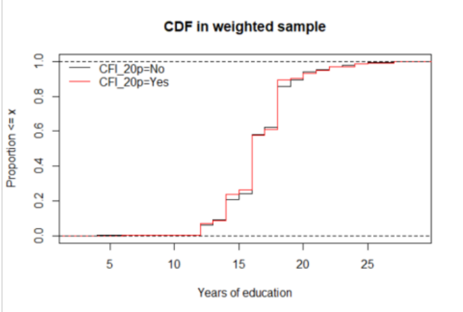

</td></tr>
</table>

Supplementary Material S2: Descriptive statistics and IPTW diagnostic findings for sensitivity analysis

To eliminate the confounding effects underlying neuropsychiatric conditions not indicative of cancer-related cognitive impairment (CRCI), we conducted a sensitivity analysis to remove all respondents who self-reported at least one of any neuropsychiatric conditions, except major depression.

Demographic and Clinical Characteristics

A total of 927 respondents did not report self-reported neuropsychiatric conditions (except major depression), with 157 (16%) remaining in the CI group, and 770 (83%) in the CNI group. The CI group, compared to CNI, comprised less non-Hispanic White respondents (77% vs 84%), had more respondents receiving chemotherapy treatment (29% vs 21%), and had self-reported more past diagnoses of major depression (17% vs 7%) (all P<0.05, **Table S1**).

IPTW diagnostic findings

IPTW was successfully at achieving covariate balance on all confounders based on the following metrics:

(1) Diagnostics based on the stabilized weights

The mean stabilized weight was close to one, at 0.979, and ranged from 0.176 to 12.439.

(2) Comparison of means and prevalences in the weighted cohort

Before IPTW, 12 of the 22 confounding variables had an SMD greater than 0.1 (**Table 1**). After IPTW weighting, differences of the 12 variables were eliminated. The highest standardized mean difference (SMD) found was 0.091 for *"Are you willing to hear about studies being conducted at the UCI campus in Irvine?"* in the weighted cohort (**Figure S1**).

(3) Comparison of higher-order moments and interactions

The interactions between the continuous variables (age and years of education), as well as SMDs of the square and cubic terms were compared in both the original unweighted and weighted samples. The square term for age, square and cubic terms

of education years, and the interaction between age and years of education in the unweighted sample reported a SMD greater than 0.1. In the weighted sample, these were corrected to be less than 0.1 (**Figure S3**).

(4) Graphical comparisons of the distribution of continuous covariates

Referring to **Figure S4**, for age at survey, the boxplots demonstrate a lower median for respondents in the CI group compared to those in the CNI group. After IPTW, the median for both groups appeared nearly equal. Similarly for years of education, the median years for the CNI group was greater than that of the CI. Again, following the IPTW, the median between both groups were nearly equal.

Table S1: Baseline characteristics of the CI and CNI groups after excluding respondents who self-reported at least one of any neuropsychiatric conditions (except major depression), prior to IPTW weighting

Variables	CNI (N=770)	CI (N=157)	Total (N=927)	p-value	SMD[a]
**CFI**					
Mean	1.59	6.72	2.46	-	-
Min, Max	0.00, 4.00	4.08, 14.00	0.00, 14.00		
**Age at survey**					
Mean (SD)	66.87 (11.29)	65.60 (12.77)	66.65 (11.56)	0.251	0.105
Median (Q1, Q3)	69.00 (61.00, 75.00)	67.00 (57.00, 75.00)	68.00 (61.00, 75.00)		
Missing, n (%)	12 (1.56)	2 (1.27)	14 (1.51)		
**Sex, n (%)**					
Male	307 (39.87)	61 (38.85)	368 (39.70)	0.858	0.021
Female	463 (60.13)	96 (61.15)	559 (60.30)		
Other	0 (0.00)	0 (0.00)	0 (0.00)		
**Race/Ethnicity, n (%)**					
Non-Hispanic White	644 (83.64)	121 (77.07)	765 (82.52)	0.047*	0.196[b]
Hispanic	29 (3.77)	9 (5.73)	38 (4.10)		
Black or African American	10 (1.30)	0 (0.00)	10 (1.08)		
Asian	21 (2.73)	11 (7.01)	32 (3.45)		
More than one population	4 (0.52)	2 (1.27)	6 (0.65)		
Refused	16 (2.08)	5 (3.18)	21 (2.27)		
Others	6 (0.78)	1 (0.64)	7 (0.76)		
Missing	40 (5.19)	8 (5.10)	48 (5.18)		
**Years of education**					
Mean (SD)	16.71 (2.43)	16.24 (2.97)	16.63 (2.54)	0.065	0.174
Median (Q1, Q3)	16.00 (16.00, 18.00)	16.00 (14.00, 18.00)	16.00 (16.00, 18.00)		
Missing, n (%)	10 (1.30)	2 (1.27)	12 (1.29)		
**Psychological Condition, n (%)**					
Major Depression	56 (7.27)	26 (16.56)	82 (8.85)	<0.001***	0.290
**Types of Cancer, n (%)**					
Skin	365 (47.40)	66 (42.04)	431 (46.49)	0.197	0.123
Breast	138 (17.92)	28 (17.83)	166 (17.91)	1.000	0.008
Genitourinary	128 (16.62)	23 (14.65)	151 (16.29)	0.555	0.061
Gynecological	68 (8.83)	12 (7.64)	80 (8.63)	0.755	0.048
Gastrointestinal	48 (6.23)	15 (9.55)	63 (6.80)	0.197	0.122
Blood and bone marrow	40 (5.19)	8 (5.10)	48 (5.18)	1.000	0.008
Head and neck (including thyroid and ocular)	32 (4.16)	8 (5.10)	40 (4.31)	0.666	0.043
Lung	19 (2.47)	10 (6.37)	29 (3.13)	0.021*	0.191
Brain and CNS	10 (1.30)	2 (1.27)	12 (1.29)	1.000	0.004
Sarcoma	8 (1.04)	4 (2.55)	12 (1.29)	0.264	0.114

**Cancer Treatment, n (%)**					
Did not receive treatment	44 (5.71)	11 (7.01)	55 (5.93)	0.577	0.053
Currently on treatment	174 (22.60)	43 (27.39)	217 (23.41)	0.221	0.115
Radiation	177 (22.99)	44 (28.03)	221 (23.84)	0.143	0.135
Chemotherapy	162 (21.04)	46 (29.30)	208 (22.44)	0.020*	0.214
Surgery	578 (75.06)	106 (67.52)	684 (73.79)	0.092	0.156
**Preferred Study Locations, n (%)**					
Are you willing to hear about studies being conducted at the UCI campus in Irvine?					
Yes	723 (93.90)	149 (94.90)	872 (94.07)	0.667	0.057
No	44 (5.71)	7 (4.46)	51 (5.50)		
Missing	3 (0.39)	1 (0.64)	4 (0.43)		
Are you willing to hear about studies being conducted at the UCI Medical Center in Orange?					
Yes	682 (88.57)	142 (90.45)	824 (88.89)	0.379	0.098
No	81 (10.52)	12 (7.64)	93 (10.03)		
Missing	7 (0.91)	3 (1.91)	10 (1.08)		

Abbreviations: CFI – Cognitive Function Instrument; CNS – central nervous system; CI – cognitively impaired; CNI – cognitively non-impaired; Q1 – quartile 1; Q3 – quartile 3; SD – standard deviation; SMD – standardized mean difference.
[a] SMD < 0.1 indicates a negligible difference in the mean or prevalence of a covariate between two groups.
[b] SMD for race and ethnicity was obtained comparing the distribution of non-Hispanic White participants and other races between the groups.
*p < 0.050.

Figure S3: Standardized mean differences in the unadjusted (original) and weighted (IPTW-weighted) cohorts, after excluding respondents who self-reported at least one of any neuropsychiatric conditions (except major depression)

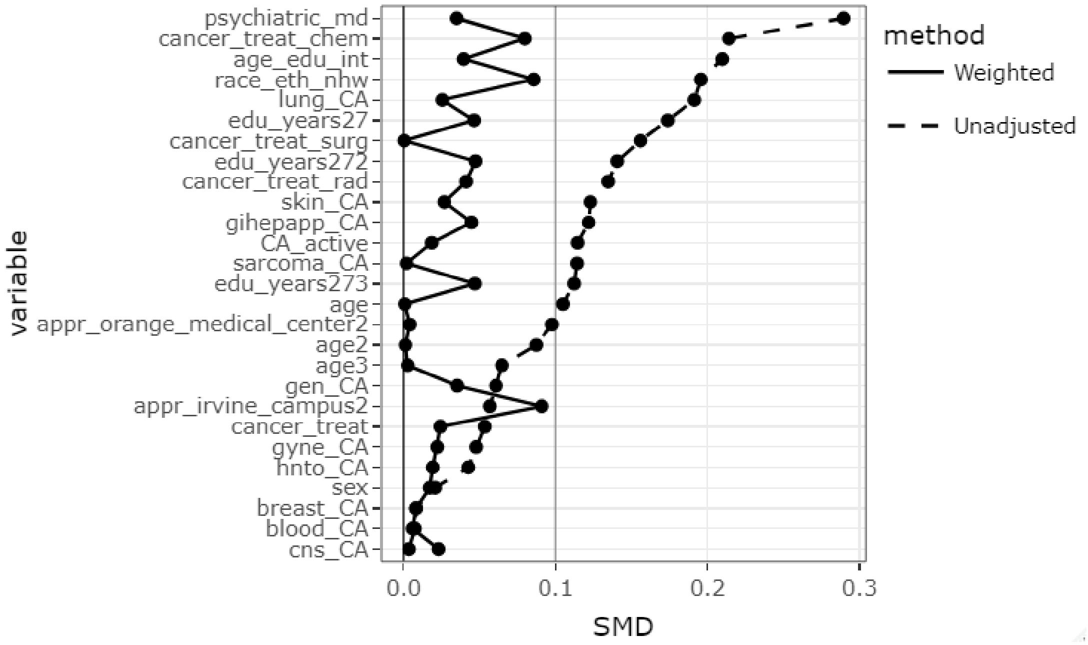

Figure S4: Distribution of age and education years between CI and CNI groups, after excluding respondents who self-reported at least one of any neuropsychiatric conditions (except major depression)

Continuous variable	Unweighted cohort	Weighted cohort
Age at survey	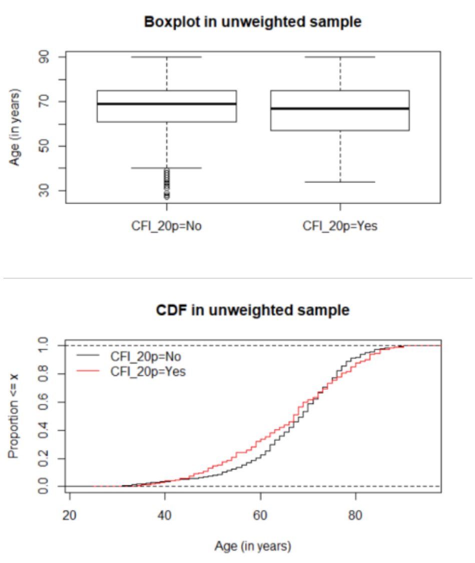	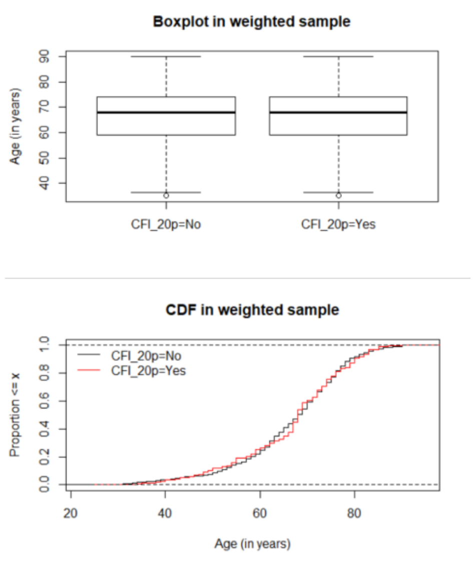
Years of education	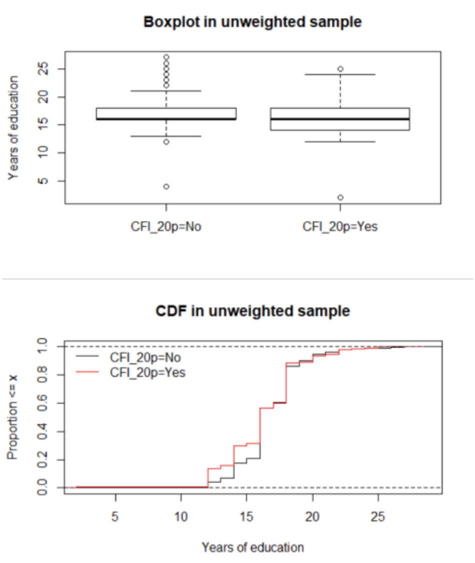	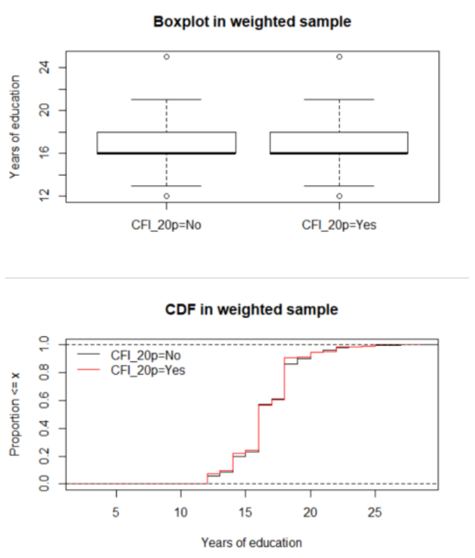

Table S2: IPTW inferential analysis – research attitudes and willingness to participate in research activities, after excluding respondents who self-reported at least one of any neuropsychiatric conditions (except major depression)

Outcomes	Non-IPTW		IPTW with propensity scores		p-value[a]
	CNI (N=770)	CI (N=157)	CNI (N=157.4)	CI (N=750.0)	
**RAQ score**					
Mean (SD)	29.06 (4.15)	28.75 (4.22)	29.06 (4.12)	28.48 (4.29)	0.151
Median (Q1, Q3)	29.00 (27.00, 32.00)	29.00 (27.00, 32.00)	29.00 (27.00, 32.00)	28.00 (27.00, 31.00)	
**Willingness to participate, n (%)**					
Are you willing to hear about studies that involve taking an approved medication?					
Yes	677 (87.92)	145 (92.36)	138.5 (87.99)	692.1 (92.28)	0.076
No	88 (11.43)	11 (7.01)	17.9 (11.37)	47.7 (6.36)	
Missing	5 (0.65)	1 (0.64)	1.0 (0.64)	10.2 (1.36)	
Are you willing to hear about studies that involve taking an investigational medication?					
Yes	612 (79.48)	136 (86.62)	125.5 (79.73)	637.1 (84.95)	0.118
No	148 (19.22)	19 (12.10)	29.9 (19.00)	97.5 (13.00)	
Missing	10 (1.30)	2 (1.27)	2.0 (1.28)	15.4 (2.05)	
Are you willing to hear about studies that involve altering your diet or lifestyle?					
Yes	710 (92.21)	148 (94.27)	145.2 (92.25)	716.6 (95.55)	0.231
No	54 (7.01)	9 (5.73)	10.9 (6.93)	33.4 (4.45)	
Missing	6 (0.78)	0 (0.00)	1.3 (0.83)	0.0 (0.00)	
Are you willing to hear about studies that involve blood draws?					
Yes	718 (93.25)	145 (92.36)	146.7 (93.20)	684.9 (91.32)	0.889
No	46 (5.97)	10 (6.37)	9.5 (6.04)	47.1 (6.28)	
Missing	6 (0.78)	2 (1.27)	1.1 (0.70)	18.0 (2.40)	
Are you willing to hear about studies that involve cognitive testing (tests of memory and thinking)?					
Yes	740 (96.10)	149 (94.90)	150.8 (95.81)	704.5 (93.93)	0.727
No	26 (3.38)	5 (3.18)	5.8 (3.68)	22.3 (2.97)	
Missing	4 (0.52)	3 (1.91)	0.8 (0.51)	23.2 (3.09)	
Are you willing to hear about studies that involve magnetic resonance imaging (MRI, a brain scan that does not involve radiation)?					
Yes	705 (91.56)	150 (95.54)	144.0 (91.49)	701.3 (93.51)	0.325
No	61 (7.92)	6 (3.82)	12.7 (8.07)	38.5 (5.13)	
Missing	4 (0.52)	1 (0.64)	0.8 (0.51)	10.2 (1.36)	
Are you willing to hear about studies that involve Positron Emission Tomography (PET, a brain scan that involves a small amount of radiation)?					
Yes	623 (80.91)	136 (86.62)	127.2 (80.81)	634.4 (84.59)	0.258
No	142 (18.44)	20 (12.74)	29.1 (18.49)	105.4 (14.05)	
Missing	5 (0.65)	1 (0.64)	1.1 (0.70)	10.2 (1.36)	
Are you willing to hear about studies that involve lumbar puncture (also known as a spinal tap)?					
Yes	286 (37.14)	77 (49.04)	59.1 (37.55)	337.8 (45.04)	0.096
No	478 (62.08)	79 (50.32)	97.1 (61.69)	402.0 (53.60)	
Missing	6 (0.78)	1 (0.64)	1.2 (0.76)	10.2 (1.36)	
Are you willing to hear about studies that involve autopsy after you die?					
Yes	537 (69.74)	121 (77.07)	109.1 (69.31)	589.4 (78.59)	0.022*
No	228 (29.61)	35 (22.29)	47.3 (30.05)	150.4 (20.05)	

Missing	5 (0.65)	1 (0.64)	1.0 (0.64)	10.2 (1.36)	
Would you be willing to visit UCI Medical Center in Orange OR the medical school in Irvine to provide a blood sample that can be used to test levels of cells, proteins, or lipids for the sake of better identifying participants for studies?					
Yes	707 (91.82)	144 (91.72)	144.9 (92.06)	685.8 (91.44)	0.791
No	62 (8.05)	13 (8.28)	12.3 (7.81)	64.2 (8.56)	
Missing	1 (0.13)	0 (0.00)	0.2 (0.13)	0.0 (0.00)	
Would you be willing to visit UCI Medical Center in Orange OR the medical school in Irvine to provide a blood sample that can be used to test for genes (DNA) for the sake of better identifying participants for studies?					
Yes	696 (90.39)	143 (91.08)	142.7 (90.66)	678.9 (90.52)	0.924
No	70 (9.09)	13 (8.28)	14.0 (8.89)	64.2 (8.56)	
Missing	4 (0.52)	1 (0.64)	0.8 (0.51)	6.9 (0.92)	
Would you be willing to receive a kit that you could use at home to provide a blood sample or swab of cells from inside your cheek to test for genes (DNA) for the sake of better identifying participants for studies?					
Yes	735 (95.45)	153 (97.45)	150.0 (95.30)	738.1 (98.41)	0.043*
No	33 (4.29)	4 (2.55)	7.1 (4.51)	11.9 (1.59)	
Missing	2 (0.26)	0 (0.00)	0.4 (0.25)	0.0 (0.00)	

Abbreviations: CFI – Cognitive Function Instrument; CI – cognitively impaired; CNI – cognitively non-impaired; IPTW – inverse probability of treatment weighting; SD – standard deviation.

[a] The p-values were computed with the IPTW-weighted sample.

*$p < 0.05$.
